# Left ventricular stroke volume decreases due to surgical procedures of anatomical lung resection

**DOI:** 10.1111/1759-7714.15434

**Published:** 2024-08-22

**Authors:** Sachie Koike, Takayuki Shiina, Keiichirou Takasuna

**Affiliations:** ^1^ Department of Thoracic Surgery Ina Central Hospital Nagano Japan; ^2^ Division of General Thoracic Surgery, Department of Surgery Shinshu University School of Medicine Nagano Japan

**Keywords:** anatomical lung resection, cardiac dysfunction, left ventricle stroke volume

## Abstract

**Objectives:**

The influence of lung resection on cardiac function has been reported, and previous studies have mainly focused on right ventricular (RV) dysfunction. As few studies have analyzed changes in left ventricular hemodynamic variables caused by lung resection, we aimed to investigate the perioperative changes in left ventricular stroke volume (LVSV) caused by anatomical lung resection.

**Methods:**

We enrolled 61 patients who underwent anatomical lung resection and perioperative LVSV monitoring. The Flo Trac system was used for dynamic monitoring. We investigated changes in LVSV after lung resection and the factors that affected these changes. The operative procedures that contributed to these changes were also investigated.

**Results:**

LVSV decreased after anatomical lung resection in the majority of patients (*n* = 38, 62.2%). Operative procedures affecting this change were (a) taping the superior pulmonary vein (SPV; right: V1‐3) before dorsal part procedure (e.g., major fissure division of right upper lobectomy, A1 + 2c, and A4 + 5 division of left upper lobectomy); (b) division of the SPV (right: V1‐3, V4 + 5); (c) division of A6‐10 (in lower lobectomy); and (d) finish division of all vessels.

**Conclusions:**

LVSV decrease was caused by anatomical lung resection in the majority of patients owing to the intraoperative procedures described above.

## Graphical abstract

Left ventricular stroke volume (LVSV) decrease caused by anatomical lung resection in the majority of patients due to intraoperative procedure described in the figure.

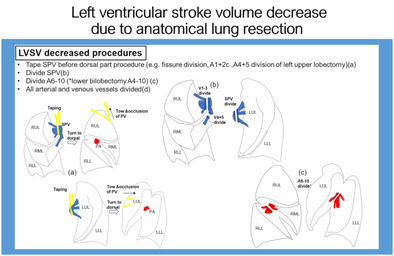

## INTRODUCTION

Lung cancer is the leading cause of worldwide cancer death,^1^ and lung resection is the gold standard treatment for early‐stage lung cancer.^2^ Lung resection offers the best chance of cure; however, it is associated with high cardiorespiratory complication rates^3^, ^4^ and significant long‐term morbidity, with many patients experiencing disabling dyspnea and decreased functional capacity, and exercise capacity.^5^, ^6^, ^7^ Several studies have reported a relationship between these problems and the reduction in pulmonary functions (e.g., forced expiratory volume in the first second [FEV1] and forced vital capacity [FVC]).^8^, ^9^, ^10^ On the other hand, in some studies, cardiac dysfunction after lung resection has been revealed, and its contribution to the reduction in function and exercise capacity was presumed.^5^, ^11^ One of these studies suggested that cardiac dysfunction is more relevant to the impairment of exercise capacity than the reduction in respiratory functions.^5^

During lung resection, we divide pulmonary arteries and veins, and these procedures may be the cause of cardiac dysfunction. Previous studies have revealed right ventricular (RV) dysfunction caused by lung resection, which has been hypothesized to result from increased RV afterload secondary to the division of proximal pulmonary artery and removal of lung tissue.^5^, ^11^, ^12^ Since these studies have mainly focused on RV dysfunction caused by lung resection, they did not clarify its influence on hemodynamic variables such as stroke volume (SV), cardiac output (CO), or cardiac index (CI), which represent the function of the cardiovascular system. Also, in these studies, the influence of pulmonary artery division was mainly studied; however, the influence of pulmonary vein division, which may affect the volume of blood flow of the left atrium and the left ventricle, has not been investigated.

In this study, we aimed to investigate perioperative changes in hemodynamic variables caused by lung resection. We have focused on perioperative changes in left ventricular stroke volume (LVSV), which has been reported on its strong correlation with exercise capacity.^13^, ^14^ We aimed to reveal (1) changes in LVSV changes after lung resection; (2) the factors that affect LVSV changes; (3) operative procedures (e.g., divide pulmonary vein, divide pulmonary artery, and divide fissure), which contribute to changes in LVSV in this study.

## PATIENTS AND METHODS

### Patients

This study was approved by the Ethics Review Board of Ina Central Hospital, Ina, Nagano, Japan (approval number: 24‐11), and the requirement for informed patient consent was waived.

We analyzed the data of patients who underwent surgery with general anesthesia in our department from June 2021 to March 2024. Of these patients, we enrolled the ones who underwent anatomical lung resection and had records of perioperative LVSV. Patients who underwent surgery other than anatomical lung resection, with missing records of perioperative LVSV, and the history of lung resection were excluded.

### Data collection of perioperative LVSV, anesthetic management, and operative approach

Perioperative LVSV data were collected using the Flo Trac system, which consists of a dedicated transducer (FloTrac Sensor, Edwards Lifesciences) connected to the radial arterial line and a Vigileo™ monitor (Edwards Lifesciences) connected to the transducer. The transducer analyzes arterial pressure waveform 20 times per second for 100 s, captures 2000 data points for analysis, and performs calculations on the data acquired during the last 20 s. The multiple indices of the cardiovascular system, such as LVSV, CO, CI, SV variation, and central venous oxygen saturation, are calculated from the data and recorded every 20 s on Vigileo™ monitor.^15^ The system was reported to be useful for monitoring intraoperative and perioperative indices of the cardiovascular system.^15^, ^16^

On the day of the operation, patients were administered general anesthesia, and the radial artery line was inserted by the anesthesiologists before the operation started. We connected the transducer and the Vigileo™ monitor to the radial artery line and started recording LVSV data. We continued recording during and after the operation and finished at 12 midnight on the day of the operation. The average value of LVSV recorded from the start of recording (monitor connected to artery line) to the start of operation was defined as preoperative LVSV (Pre LVSV). The average value of LVSV recorded from the end of the operation to 12 midnight was defined as postoperative LVSV (Post LVSV). In addition, intraoperative LVSV was checked to investigate LVSV changes caused by the operative procedures.

The fluid volume infused before the start of the operation was 500 mL in all the enrolled patients, who underwent video‐assisted thoracic surgery (VATS).

### Assessment

We compared Pre LVSV with Post LVSV to identify the changes in LVSV after lung anatomical resection. We collected the data of age, sex, body surface area (BSA), cardiovascular comorbidities, type of surgery, intraoperative/postoperative fluid volume, operative time, bleeding, cardiovascular complications, and histology of enrolled patients to investigate the factors that affect Post LVSV change. We also checked the records of LVSV during operation, videos of operation, and anesthetic records and investigated the influence of each operative procedure on LVSV changes.

### Statistical analysis

Continuous variables are described as median and interquartile intervals. Categorical variables are described as the number of cases and percentages. Mann–Whitney *U* tests were used to compare the categorical variables between independent groups, and Pearson's chi‐squared test was used to compare the categorical variables in independent groups.

Differences were considered statistically significant at *p* < 0.05.

All statistical analyses were performed using the Statistical Package for the Social Sciences (SPSS) software (version 26.0; SPSS, IBM, USA).

## RESULTS

### Patient characteristics

**FIGURE 1:**
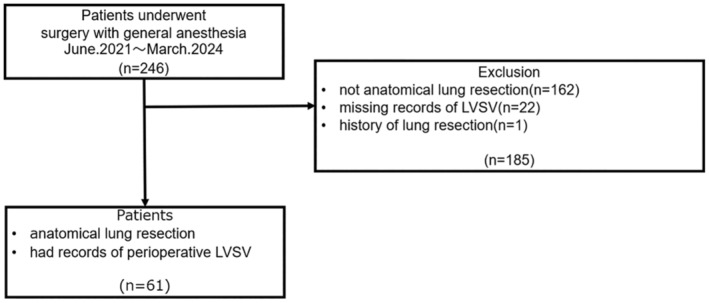
Schema of the study cohort. LVSV, left ventricular stroke volume.

A total of 246 patients underwent surgery with general anesthesia in our department from June 2021 to March 2024. Of these patients, 61 patients who underwent anatomical lung resection met our inclusion criteria (Figure 1). The patient characteristics are shown in Table 1. Of the enrolled patients, 33 (54.1%) were male, with a median age of 72 years. There were 9 patients (14.8%) who had cardiovascular comorbidities. The number of patients who underwent right upper lobectomy was the largest of all types of resection (47.5%), followed by right lower lobectomy (14.8%) and left upper lobectomy (14.8%). The number of enrolled patients who underwent segmentectomy was two, which are right basal segmentectomy and left S6 segmentectomy.

**TABLE 1:** Patient characteristics.

Variable	Value
Preoperative factors	
Sex (male)	33 (54.1)
Age (years)	72 (64–74.5)
BSA (m^2^)	1.63 (1.47–1.76)
Cardiovascular comorbidities	9 (14.8)
Resection type	
Right upper lobectomy	29 (47.5)
Right middle lobectomy	7 (11.5)
Right lower lobectomy	9 (14.8)
Right middle and lower lobectomy	1 (1.6)
Left upper lobectomy	9 (14.8)
Left lower lobectomy	4 (6.6)
Segmentectomy	2 (3.3)
Left sided/right sided	14 (23.0)
No. of resected segment	3 (3–4)
Intraoperative factors	
Operative time (min)	199 (155–256)
Bleeding (mL)	24 (12–61)
Intraoperative fluid volume (mL)	1350 (1150–1590)
Postoperative factors	
Postoperative fluid volume (mL)	1912 (1772–2180)
Cardiovascular complications	5 (8.2)
Histology	
Lung cancer	54 (88.5)
Others	7 (11.5)

Abbreviations: BSA, body surface area; No., number.

### Changes in LVSV after anatomical lung resection and affected factors

Post LVSV decreased compared with Pre LVSV in 38 patients (62.2%), while 23 patients (37.7%) had almost the same or increased Post LVSV compared with Pre LVSV. Analyzed in all enrolled patients, Post LVSV significantly decreased compared with Pre LVSV (*p* = 0.01).

Patient characteristics were compared between the LVSV‐decreased group and the non‐decreased group (Table 2). The proportion of males in the non‐LVSV‐decreased group was significantly larger than the LVSV‐decreased group (*p* = 0.016), and this is the only significant difference between the two groups. The median age and BSA were almost the same in the two groups. The rate of cardiovascular comorbidities was higher in the LVSV‐decreased group, but it was not significant (18.4% vs. 8.4%, *p* = 0.30). The proportion of the type of resection undergone by the patients in both groups was not significantly different; however, the proportion of the right middle lobe and the right lower lobe was relatively larger in the LVSV‐decreased group (right middle lobe 15.8% vs. 4.3%, right lower lobe 18.4% vs. 8.7%). Two enrolled patients who underwent segmentectomy were in the non‐LVSV‐decreased group. The number of resected segments was not significantly different between the LVSV‐decreased group and the non‐LVSV‐decreased group. Operative time, amount of bleeding, intraoperative fluid volume, and postoperative fluid volume were almost the same in the two groups. The cardiovascular complication rate was more than twice in the LVSV‐decreased group (10.3% vs. 4.3%).

**TABLE 2:** Comparison of patient characteristics between the LVSV‐decreased group and the non‐decreased group.

Variable	LVSV decrease		*p* value
	Yes (*n* = 38)	No (*n* = 23)	
Preoperative factors			
Sex (male)	16 (42.1)	17 (73.9)	0.016
Age (years)	72 (62.8–75)	72 (65–74)	0.81
BSA (m^2^)	1.62 (1.46–1.73)	1.7 (1.48–1.87)	0.15
Cardiovascular comorbidities	7 (18.4)	2 (8.7)	0.3
Resection type			0.22
Right upper lobectomy	15 (39.5)	14 (60.9)	0.1
Right middle lobectomy	6 (15.8)	1 (4.3)	0.17
Right lower lobectomy	7 (18.4)	2 (8.7)	0.3
Right middle and lower lobectomy	1 (2.6)	0 (0.0)	0.43
Left upper lobectomy	6 (15.8)	3 (13.0)	0.77
Left lower lobectomy	3 (7.9)	1 (4.3)	0.59
Segmentectomy	0 (0.0)	2 (8.7)	0.07
Left sided/right sided	9 (23.7)	5 (21.7)	0.86
No. of resected segment	3 (3–4)	3 (3–4)	0.55
Intraoperative factors			
Operative time (min)	201 (157–261)	187 (142–253)	0.28
Bleeding (mL)	27 (15–77)	23 (10–50)	0.29
Intraoperative fluid volume (mL)	1350 (1138–1663)	1400 (1150–1580)	0.7
Postoperative factors			
Postoperative fluid volume (mL)	1903 (1787–2156)	2028 (1731–2259)	0.7
Cardiovascular complications	4 (10.3)	1 (4.3)	0.39
Histology			0.17
Lung cancer	32 (84.2)	22 (95.7)	
Others	6 (15.8)	1 (4.3)	

Abbreviations: BSA, body surface area, LVSV, left ventricular stroke volume; No. = number.

### LVSV‐decreased procedures

**FIGURE 2:**
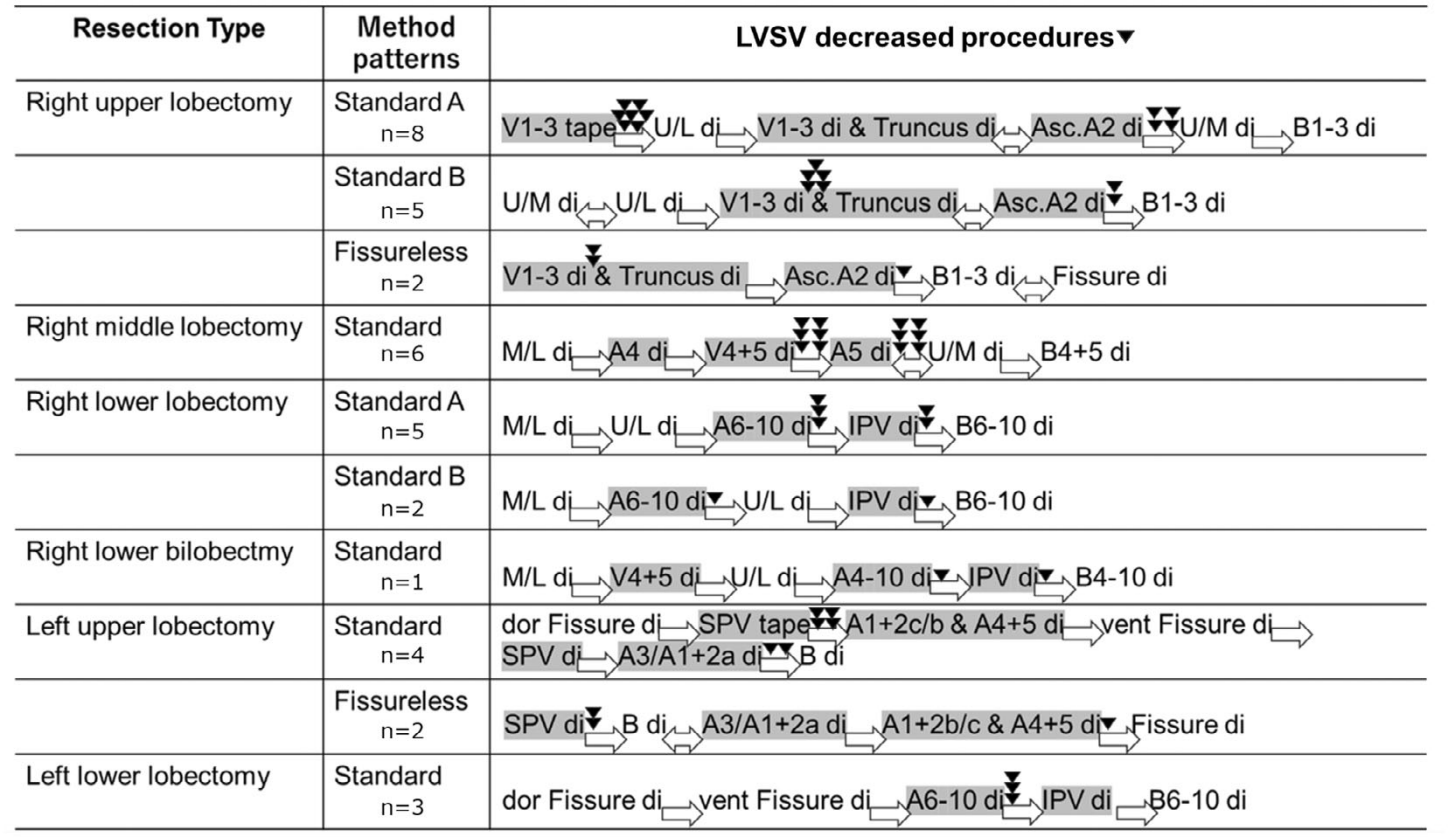
Intraoperative LVSV‐decreased procedures. The timing of LVSV decrease in each patient was described by the black arrow. LVSV decreased more than once in several patients. A, artery; Asc.A2, ascending A2, A2b; B, bronchus; di, division; dor, dorsal; IPV, inferior pulmonary vein, LVSV, left ventricular stroke volume; M/L di, division of major fissure between the middle and lower lobes; SPV, superior pulmonary vein; Truncus, truncus superior; U/L di, division of major fissure between the upper and lower lobes; U/M di, division of minor fissure; V, vein; vent, ventral.

We investigated the intraoperative LVSV records of all 38 LVSV‐decreased patients and identified the LVSV‐decreased procedures. The LVSV‐decreased procedure of each type of lung resection is described in Figure 2. Each type of lung resection can be classified into several patterns of methods, and the LVSV‐decreasing procedure varies with each pattern of method.

Right upper lobectomy patients (*n* = 15) can be classified into three patterns of methods as follows:

- Standard A (*n* = 8): Started with V1‐3 taping. After that, the major fissure between the upper and lower lobes was divided. And then, veins and arteries were divided. After that, the minor fissure was divided. Finally, the bronchus of the right upper lobe was divided.
- Standard B (*n* = 5): Started with the division of fissure. V1‐3 and arteries were divided after fissure division, and finally, the bronchus was divided.
- Fissureless (*n* = 2): For patients whose fissure was incomplete. V1‐3 and arteries were divided first, and then, the bronchus was divided. The fissure was divided at the end of the resection.

During the Standard A method, LVSV decreased after V1‐3 taping in most of the patients and after dividing all the vessels in some patients. On the other hand, during Standard B methods, LVSV decreased when V1‐3 was divided in most of the patients, and after all, the vessels were divided in some patients. Similar to the Standard B method, LVSV decreased when V1‐3 was divided in most of the patients, and after all, the vessels were divided in some patients during the fissureless method.

The methods were almost the same in all of the patients who underwent right middle lobectomy (*n* = 6). First, the major fissure was divided, and then A4 and V4 + 5 were divided. Then, A5 or minor fissure was divided. At the end of the resection, B4 + 5 was divided. LVSV decreased after V4 + 5 was divided, and all the vessels were divided.

Patients who underwent right lower lobectomy (*n* = 7) can be classified into two method groups as follows:

- Standard A (*n* = 5): The major fissure was divided first. Then, A6‐8 was divided. After that, the inferior pulmonary vein (lPV) was divided. Finally, the bronchus was divided.
- Standard B (*n* = 2): The major fissure between the upper lobe and the lower lobe was divided first. Then, A6‐8 was divided. After that, the major fissure between the middle lobe and the lower lobe was divided; following the division of the IPV, the bronchus was divided.

LVSV decreased after the division of A6‐8 in most of the patients in both methods. In some patients, LVSV also decreased after all vessels were divided.

Only one patient underwent right lower bilobectomy, in whom the LVSV decreased after A4‐10 was divided, and all the vessels were divided.

Left upper lobectomy patients (*n* = 6) can be classified into two groups of methods as follows:

- Standard (*n* = 4): First, the dorsal part of the fissure was divided. Then, the superior pulmonary vein (SPV) was taped; after that, A1 + 2c, A1 + 2b, and A4 + 5 were divided. Following the division of the ventral part of the fissure, SPV, A1 + 2a, and A3 were divided. The bronchus was divided at the end of the resection.
- Fissureless (*n* = 2): First, the SPV was divided. Then, the bronchus was divided, and after that, the pulmonary arteries were divided; finally, the fissure was divided.

During the standard method, LVSV decreased after SPV taping in most of the patients. In some patients, LVSV decreased after all the vessels were divided. On the other hand, during the fissureless method, LVSV decreased after SPV division in all two patients. LVSV also decreased after division of all vessels in one patient.

All of the left lower lobectomy patients (*n* = 3) were operated using the same method. The fissure was divided first, and then A6‐10 was divided. Following the division of the IPV, the bronchus was divided. LVSV decreased after the division of A6‐10 in all patients.

**FIGURE 3:**
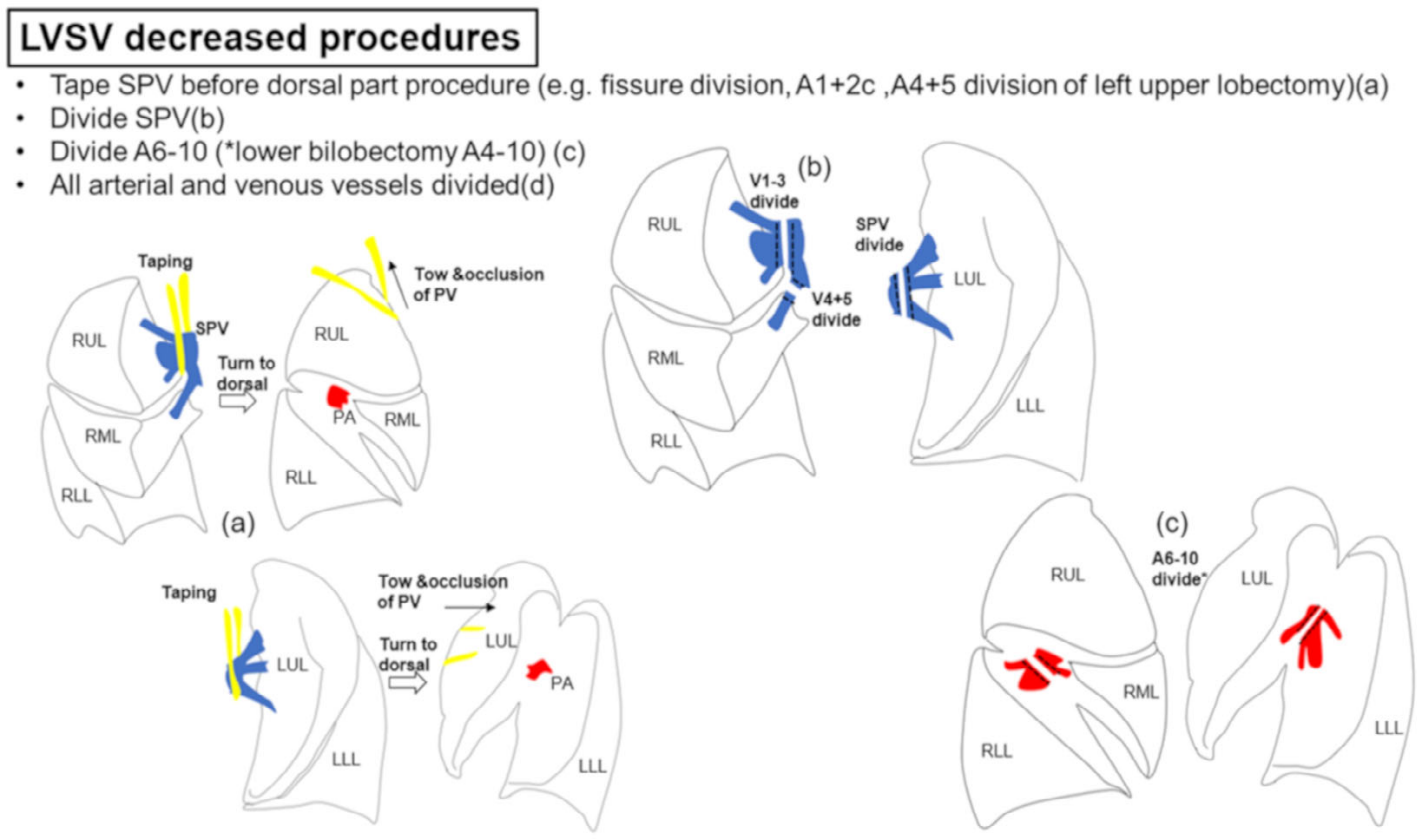
Summarized result of intraoperative LVSV‐decreased procedures. LLL, right lower lobe; LUL, left upper lobe; LVSV, left ventricular stroke volume; PA, pulmonary artery; PV, pulmonary vein; RLL, right lower lobe; RML, right middle lobe; RUL, right upper lobe; SPV, superior pulmonary vein; UL, right upper lobe; VSV, left ventricular stroke volume.

To summarize the results of all methods, the LVSV‐decreased procedure can be described as follows: (a) taping the SPV (right: V1‐3) before the dorsal part procedure (e.g., major fissure division of right upper lobectomy, A1 + 2c, and A4 + 5 division of left upper lobectomy); (b) division of the SPV (right V1‐3 and V4 + 5); (c) division of A6‐10 (in lower lobectomy); and (d) finish division of all vessels (Figure 3).

We also investigated the intraoperative LVSV records of 23 non‐LVSV‐decreased patients. LVSV of patients who underwent lobectomy also decreased after (a)~(d) procedures, as mentioned above. However, the LVSV of those patients increased again to the original amount or more during operation (*n* = 20) and after operation (*n* = 3). The LVSV of patients who underwent right basal segmentectomy decreased after the A7‐10 resected, but it recovered to the original amount during operation. LVSV of left S6 segmentectomy did not decrease during operation.

## DISCUSSION

While most of previous studies about the perioperative cardiac function of lung resection focused on RV dysfunction,^5^, ^11^, ^12^ this study investigated LVSV changes caused by anatomical lung resection. The main findings of this study are as follows: (1) we found that LVSV decreased after anatomical lung resection in the majority of patients, and the female sex might be its risk factor; and (2) LVSV‐decreased procedure during operation was (a) taping the SPV (right: V1‐3) before the dorsal part procedure (e.g., major fissure division of right upper lobectomy, A1 + 2c, and A4 + 5 division of left upper lobectomy); (b) division of the SPV (right: V1‐3 and V4 + 5); (c) division of A6‐10 (in lower lobectomy); and (d) finish division of all vessels.

In this study, we have investigated the intraoperative LVSV decreasing procedures using a semi‐invasive dynamic fluid monitoring system (FloTrac, Edwards Lifesciences). No other studies investigate the intraoperative LVSV changes in each type of lung resection, and these are the first data. In many previous studies, the division of pulmonary artery has been considered as one of the leading causes of cardiac dysfunction, which mainly affects RV function.^5^, ^11^, ^12^ However, in our study about LVSV, two operative procedures related to the pulmonary vein were identified as a cause of LVSV decrease. One is (a) taping the SPV (right: V1‐3) before the dorsal part procedure, and another is (b) division of the SPV (right: V1‐3 and V4 + 5). Although no vessels were divided with the procedure (a), we presumed that taped vessels were towed and occluded when we approached the dorsal part of the lung with the taping, and this might be the similar condition to SPV division (Figure 3a). These results revealed that blood flow reduction in the SPV caused by division or occlusion results in LVSV decrease. Some previous studies reported the relationship between pulmonary venous flow and the function of left ventricles.^17^, ^18^, ^19^ In addition, Struß N, et al. suggested that compression of the pulmonary veins reduces the preload of the left heart and causes the left heart decompensation.^20^ These reports may be the explanation of the results of our study. Even the division of V4 + 5 of the right middle lobe, which seems to have a small amount of blood flow with a small diameter, affected the LVSV decrease in our study, so we should be careful when we divide the SPV or its branches. Regarding the patients who underwent lower lobectomy, A6‐10 division was the main cause of LVSV decrease in our study, while the division of pulmonary arteries of the upper or middle lobe had few effects on LVSV changes. We assumed that the difference in the diameter of the arteries may affect this result. The diameter of A6‐10 was quite larger than that of pulmonary arteries of the upper or middle lobe (e.g., right A2b, truncus superior, left A3, A4, and A5). Division of arteries with a larger diameter may result in higher volume loss of venous return to the left atrium. This might affect the amount of LVSV decrease after pulmonary artery division. In contrast to the SPV, the division of the IPV in lower lobectomy had a smaller influence on LVSV decrease. In all the patients who underwent lower lobectomy, division of the IPV occurred after A6‐10 division. We assumed that this order of procedure may affect the result. After the A6‐10 division, little blood flow may be supplied to the IPV, and this may be the cause of a poor effect on the IPV.

We found 38 patients who represented decreased Post LVSV compared with Pre LVSV due to intraoperative procedures as described above (LVSV‐decreased group), and 23 patients whose LVSV recovered from intraoperative LVSV decreased and represented almost the same or increased amount of LVSV postoperatively (non‐LVSV‐decreased group). We compared the characteristics of the two groups to investigate the factors that affect the difference. The number of female sex was significantly larger in the LVSV‐decreased group, and this was the only significant difference between the two groups. We suspected the influence of physique and related perioperative fluid volume; however, BSA and perioperative fluid volume were almost the same in both groups. There are no clear explanations for this difference.; however, some of the previous studies explained the difference in LVSV reaction to stress between male and female. In those studies, the amount of LVSV increased after laboratory stress was greater in male.^21^, ^22^ This reaction difference between sexes might be one of the factors that affect the difference in LVSV recovery from the decrease caused by lung resection. We have considered that several perioperative factors, such as intraoperative fluid volume (fluid volume before the start of operation was the same in all patients), operative time (may affect intraoperative fluid volume), bleeding, and postoperative fluid volume, which seemed to change preload affect the LVSV changes; however, these factors of both groups were similar. The rate of cardiovascular comorbidities and cardiovascular complications was higher in the LVSV‐decreased group but not significant. Increasing the sample size may clarify the effects of LVSV changes.

We hypothesized that a larger number of resected segments contributed to Post LVSV decrease (e.g., more right lower lobectomy and less right middle lobectomy in the LVSV‐decreased group); however, a median number of resected segments was almost the same in both groups. Also, contrary to our hypothesis, a portion of the right middle lobectomy, which consists of the resection of only two segments, was quite larger in the LVSV‐decreased group (18.4% vs. 8.4%). This result may affect the result of poor effect of resected segments number on Post LVSV decrease. Several studies have reported negative influences of middle lobe removal on postoperative mortality and morbidity of advanced lung cancer.^23^, ^24^ They revealed the unexpected significant influence of middle lobe removal, and this result perhaps related to the result of our study about LVSV decrease in the right middle lobectomy. On the other hand, two patients who underwent segmentectomy were in the non‐LVSV‐decreased group. The patient who underwent right basal segmentectomy represented almost the same intraoperative LVSV changes as right lower lobectomy and the amount of LVSV recovered during the operation. Unlike these changes, the patient who underwent left S6 segmentectomy had no LVSV‐decreased procedure. Further data are required to examine the effect of segmentectomy on LVSV changes and to clarify the LVSV reserve effect of single‐segment resection.

This study had several limitations. First, the number of patients was relatively small, because the LVSV data were missing. The number of patients with each type of resection other than right upper lobectomy was quite small, and only two enrolled patients underwent segmentectomy. This may affect the accuracy of the result of the relationship between Post LVSV decrease and the number of resected segments or type of resection. Second, this was a single‐institution study. Third, we investigated only perioperative changes in LVSV and have no data about long‐term results. Further study about the long‐term influence of anatomical lung resection on LVSV might be needed. Finally, we did not investigate the exact influence of LVSV decrease on functional or exercise capacity after anatomical lung resection.

## CONCLUSION

In conclusion, we found that LVSV decreased after anatomical lung resection in the majority of patients. LVSV decrease was caused by surgical procedures as follows: (a) taping the SPV (right: V1‐3) before the dorsal part procedure (e.g., major fissure division of right upper lobectomy, A1 + 2c, and A4 + 5 division of left upper lobectomy); (b) division of the SPV (right: V1‐3 and V4 + 5); (c) division of A6‐10 (in lower lobectomy); and (d) finish division of all vessels. We should avoid (a) taping the SPV before the dorsal part procedure to reduce the influence on LVSV. Also, we should take care of hemodynamic changes after procedures (b)–(d), which cannot be avoided to complete lobectomy.

## AUTHOR CONTRIBUTIONS

All authors: Conception, design, collection, manuscript writing, final approval of manuscript, and assembly of data. Sachie Koike: Data analysis and interpretation.

## FUNDING INFORMATION

Nothing to declare.

## CONFLICT OF INTEREST STATEMENT

There are no conflicts of interest to declare.

## Data Availability

The data underlying this article cannot be shared publicly for protecting privacy of individuals that participated in this study. The data may be shared on reasonable request to the corresponding author after an additional approval by the Institutional Review Board of Ina Central Hospital, Ina, Nagano, Japan.
